# *MTO1* Mutations are Associated with Hypertrophic Cardiomyopathy and Lactic Acidosis and Cause Respiratory Chain Deficiency in Humans and Yeast

**DOI:** 10.1002/humu.22393

**Published:** 2013-09-17

**Authors:** Enrico Baruffini, Cristina Dallabona, Federica Invernizzi, John W Yarham, Laura Melchionda, Emma L Blakely, Eleonora Lamantea, Claudia Donnini, Saikat Santra, Suresh Vijayaraghavan, Helen P Roper, Alberto Burlina, Robert Kopajtich, Anett Walther, Tim M Strom, Tobias B Haack, Holger Prokisch, Robert W Taylor, Ileana Ferrero, Massimo Zeviani, Daniele Ghezzi

**Affiliations:** 1 Department of Life Sciences, University of ParmaParma, Italy; 2 Unit of Molecular Neurogenetics, Fondazione IRCCS (Istituto di Ricovero e Cura a Carattere Scientifico) Istituto Neurologico “CarloBesta”Milan, Italy; 3 Wellcome Trust Centre for Mitochondrial Research, Institute for Ageing and Health, Newcastle UniversityNewcastle upon Tyne, UK; 4 Department of Clinical Inherited Metabolic Disorders, Birmingham Children's Hospital NHS Foundation TrustBirmingham, UK; 5 Department of Child Health, Heart of England NHS Foundation TrustBirmingham, UK; 6 Division of Inborn Errors of Metabolism, Department of Paediatrics, University HospitalPadua, Italy; 7 Institute of Human Genetics, Helmholtz Zentrum MünchenNeuherberg, Germany; 8 Institute of Human Genetics, Technische Universitat MünchenMunich, Germany; 9 MRC Mitochondrial Biology UnitCambridge, UK

**Keywords:** MTO1, hypertrophic cardiomyopathy, lactic acidosis, mitochondrial disorder, yeast

## Abstract

We report three families presenting with hypertrophic cardiomyopathy, lactic acidosis, and multiple defects of mitochondrial respiratory chain (MRC) activities. By direct sequencing of the candidate gene *MTO1*, encoding the mitochondrial-tRNA modifier 1, or whole exome sequencing analysis, we identified novel missense mutations. All *MTO1* mutations were predicted to be deleterious on MTO1 function. Their pathogenic role was experimentally validated in a recombinant yeast model, by assessing oxidative growth, respiratory activity, mitochondrial protein synthesis, and complex IV activity. In one case, we also demonstrated that expression of wt *MTO1* could rescue the respiratory defect in mutant fibroblasts. The severity of the yeast respiratory phenotypes partly correlated with the different clinical presentations observed in *MTO1* mutant patients, although the clinical outcome was highly variable in patients with the same mutation and seemed also to depend on timely start of pharmacological treatment, centered on the control of lactic acidosis by dichloroacetate. Our results indicate that *MTO1* mutations are commonly associated with a presentation of hypertrophic cardiomyopathy, lactic acidosis, and MRC deficiency, and that ad hoc recombinant yeast models represent a useful system to test the pathogenic potential of uncommon variants, and provide insight into their effects on the expression of a biochemical phenotype.

## Introduction

Mitochondrial disorders are a group of syndromes associated with severe dysfunction of oxidative phosphorylation (OXPHOS), the main energy bioreactor of cells. Cardiomyocytes, with their extremely high request of energy, are one of the major targets of OXPHOS impairment, and infantile hypertrophic cardiomyopathy is a key clinical feature in many mitochondrial disorders. We have recently reported the first patients affected by hypertrophic cardiomyopathy and lactic acidosis carrying mutations in *MTO1* (MIM #614702) [Ghezzi et al., 2012]. Two were siblings, compound heterozygous for c.1858dup (p.Arg620Lysfs*8) and c.1282G>A (p.Ala428Thr) mutations, who died in their first days of life due to sudden bradycardia. Muscle and fibroblasts showed decreased activities of mitochondrial respiratory chain (MRC) complex I (CI) and CIV. The third patient, homozygous for the c.1282G>A (p.Ala428Thr) mutation, had also early-onset cardiac hypertrophy with severe lactic acidosis, and defective CI + CIV activities in muscle; however, he dramatically improved on a permanent treatment with dichloroacetate (DCA) and cofactors, being now 20 years old with compensated, stable hypertrophic cardiomyopathy.

*MTO1* (MIM #614667), a gene conserved in all eukaryotes, encodes one of the two subunits of the enzyme that catalyzes the 5-carboxymethylaminomethylation (mnm5s2U34) of the wobble uridine base in the mitochondrial tRNAs specific to Gln, Glu, Lys, Leu(UUR), and possibly Trp [Suzuki et al., 2011; Wang et al., 2010]. The other subunit is encoded by *MSS1* in yeast and *GTPBP3* in humans (MIM #608536). For mt-tRNAs for Gln, Glu, Lys, this modification is usually coupled to the 2-thiolation of the same uridine moiety, a reaction catalyzed by 2-thiouridylase, encoded by yeast *MTO2* or human *TRMU* (MIM #610230). Both these posttranscriptional modifications increase accuracy and efficiency of mitochondrial DNA (mtDNA) translation by influencing tRNA structure, binding to the ribosome, stabilization of the correct codon-anticodon pairing [Kurata et al., 2008; Murphy et al., 2004; Takai, 2005; Umeda et al., 2005; Urbonavicius et al., 2001; Wang et al., 2010; Yarian et al., 2000; Yasukawa et al., 2001], and tRNA recognition by the cognate aminoacyltransferase [Krüger and Sørensen, 1998; Sylvers et al., 1993].

In our previous work, we investigated the functional consequences of the *MTO1* mutations in a simple eukaryotic model system, *Saccharomyces cerevisiae* [Ghezzi et al., 2012]. The analysis was performed mainly in a mutant yeast strain harboring a C>G transversion at nucleotide 1,477 of the 15S rRNA mtDNA gene [Colby et al., 1998], which results in a synthetic phenotype with *MTO1* disruption. The mutation disrupts the C1477–G1583 base pairing in a functionally relevant hairpin structure, which is part of the decoding site (site A) of the ribosome, where codon–anticodon recognition takes place [Yan et al., 2005]. This mutation confers resistance to the antibiotic paromomycin by destabilizing the hairpin. We chose this strain because the human mitochondrial 12S rRNA contains a hairpin structure that corresponds to the paromomycin-resistant variant in yeast. We showed that the yeast Ala431Thr change, corresponding to human Ala428Thr, reduced mitochondrial respiratory activity, whereas the mutation equivalent to human Arg620Lysfs*8 behaved as a null allele.

We present here the identification of five additional *MTO1* mutant subjects (two couples of siblings, and a sporadic case) who also present with hypertrophic cardiomyopathy and lactic acidosis, thus, strengthening a consistent genotype/phenotype correlation. We confirm the pathogenic role of the two novel mutations in the yeast model and, for the milder variant, by complementation studies in mutant fibroblasts.

## Materials and Methods

### Patients

Informed consent for participation in this study was obtained from the parents of all patients, in agreement with the Declaration of Helsinki and approved by the Ethical Committees of the Institutes participating in this study, where biological samples were obtained.

**Figure 1:**
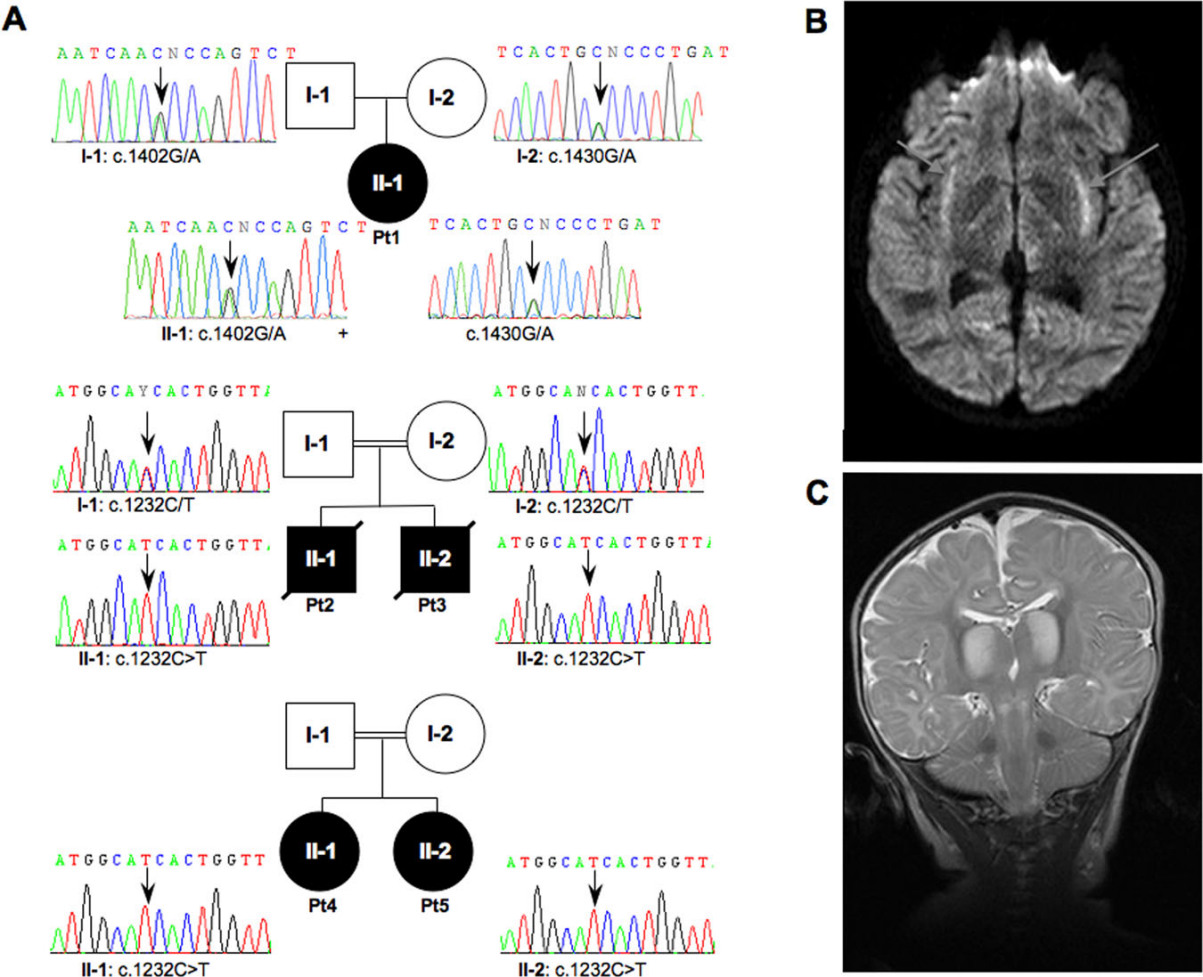
Pedigrees and radiological features. A: Pedigrees and electropherograms of the *MTO1* genomic region encompassing the nucleotide substitutions in patients and available parents. Black symbols designate affected subjects. B: Brain MRI of Pt1. Transverse FLAIR image showing abnormal hyperintensity in the region of the claustrum and surrounding capsulae (arrows). C: Brain MRI of Pt2. Coronal T2-weighted sequence showing abnormal hyperintense signals of the thalami and diffusely abnormal signal in the subcortical white matter. Lesions are also present in the brainstem. The cerebellar folia are normal.

We studied a first cohort of 30 patients with cardiomyopathy and a biochemical defect of the MRC, affecting either CI alone or multiple complexes, and a second small group of four cases with isolated CIV deficiency and at least one affected sibling, irrespective of their clinical presentations (ranging from cardiomyopathy to encephalopathy). Table 1 summarizes the main clinical, laboratory and biochemical features of five patients from three families (Fig. 1A) with *MTO1* mutations. All these subjects showed early-onset, progressive hypertrophic cardiomyopathy, and lactic acidosis. Some did also display neurological features affecting the peripheral or the central nervous system, or both, associated with neuropathological abnormalities documented by MRI (Fig. 1B and C). Detailed case reports are described in the Supporting Information.

**Table 1:** Clinical Synopsis and Biochemical Features of MTO1 patients

Patient		Familiarity	Gender	Age of onset	Relevant clinical features	Actual age/Outcome	Cause of death	Metabolic findings	Biochemical MRC defects	Mutations in *MTO1*
#1	Pt1, present study	No	F	2 days	Psychomotor delay, hypotonia, dystonia. Later, hypertrophic cardiomyopathy.	14 yrs	–	Lactic acidemia, hyperalaninemia	Ms: ↓ CI and CIVFbs: ↓ MRR	p.[Ala428Thr]; [Arg477His]
#2	Pt2, present study	Brother of #3; consanguineous parents	M	Birth	Poor feeding due to swallowing difficulties.Failure to thrive. Later, hypertrophic cardiomyopathy. Aspiration pneumonia. Hypotonia.	+12 mo	Cardio-respiratory arrest.	Hypoglycaemia, lactic acidemia	Ms: ↓ CI and CIVFbs: ↓ MRR	p.[Thr411Ile];[Thr411Ile]
#3	Pt3, present study	Brother of #2; consanguineous parents	M	Birth	Poor feeding due to swallowing difficulties. Failure to thrive. Early-onset hypertrophic cardiomyopathy. Hypotonia.	+3 mo	n.d.	Lactic acidemia	n.d.	p.[Thr411Ile];[Thr411Ile]
#4	Pt4, present study	Sister of #5; consanguineous parents	F	3 mo	Early-onset hypertrophic cardiomyopathy. Bronchiolitis-like illness. Encephalopathy and seizures.	19 yrs	–	Lactic acidemia	Ms: ↓ CIV	p.[Thr411Ile];[Thr411Ile]
#5	Pt5, present study	Sister of #4; consanguineous parents	F	5 mo	Upper respiratory illness. Hypertrophic cardiomyopathy and WPW. Psychomotor delay.	12 yrs	–	Lactic acidemia, hyperalaninemia, ketonuria	Ms: ↓ CIV	p.[Thr411Ile];[Thr411Ile]
#6	Pt1, Ghezzi et al., (2012)	Brother of #7	M	Birth	Hypertrophic cardiomyopathy.	+19 days	Sudden bradycardia	Lactic acidemia, hyperalaninemia	Ms: ↓ CI and CIVFbs: ↓CIII and CIV; ↓ MRR	p.[Ala428Thr];[Arg620Lysfs^*^8]
#7	Pt2, Ghezzi et al., (2012)	Sister of #6	F	Birth	Hypertrophic cardiomyopathy with tachycardia. Hypotonia.	+40 days	Sudden bradycardia	Lactic acidemia	Ms: ↓ CI and CIVFbs: ↓ CI; ↓ MRR	p.[Ala428Thr];[Arg620Lysfs^*^8]
#8	Pt3, Ghezzi et al., (2012)	No	M	1 mo	Weakness, lack of ocular fixation. Hypertrophic cardiomyopathy with sinus bradycardia. Moderate bilateral optic atrophy.	20 yrs	–	Lactic acidemia	Ms: ↓ CI and CIV	p.[Ala428Thr];[Ala428Thr]

Ms, muscle biopsy; Fbs, fibroblasts; MRC, mitochondrial respiratory chain; CI–CIV, complexes I–IV; WPW, Wolff–Parkinson–White syndrome.

### Molecular Analysis

Genomic DNA was extracted by standard methods. Exons and exon–intron boundaries of human *MTO1* (NM_012123.3; NP_036255) were amplified using primers listed in Supp. Table S1, and analyzed by Sanger sequencing. Whole-exome next-generation sequencing (WES) and variant filtering were performed as described [Ghezzi et al., 2012]. Nucleotide numbering reflects cDNA numbering with +1 corresponding to the A of the ATG translation initiation codon in the reference sequence, according to journal guidelines (www.hgvs.org/mutnomen). The initiation codon is codon 1. All variants reported have been submitted to LSDB (http://www.lovd.nl/MTO1).

### Biochemical Assays

The activities of MRC complexes and citrate synthase in muscle homogenates were measured as described [Bugiani et al., 2004]. Microoxygraphy was used to measure maximal respiration rate (MRR), spare respiratory capacity (SRC), respiratory control ratio, and oxygen consumption rate (OCR)/extracellular acidification rate (ECAR) in fibroblasts, using SeaHorse FX-24 or FX-96 [Invernizzi et al., 2012]. For transduced cells, F14 medium (Euroclone), supplemented with EGF, FGF, insulin, and uridine, was used instead of DMEM.

### In Silico Analysis

The pathogenicity of the human mutations was predicted by using five bioinformatic tools based on heuristic methods: PANTHER (http://www.pantherdb.org), SIFT (http://sift.jcvi.org), PolyPhen-2 (http://genetics.bwh.harvard.edu/pph2), SNPs&GO (http://snps-and-go.biocomp.unibo.it/snps-and-go), and MutPred (http://mutpred.mutdb.org). Structural analysis was performed using the structure of *Chlorobium tepidum* GidA (PDB ID 3CP8 at http://www.rcsb.org/pdb/home/home.do). Models of mutant proteins were constructed using SwissModel (http://swissmodel.expasy.org/) and superimposed with Swiss-Pdb Viewer Magic fit tool. Protein regions were visualized by the RasMol software package.

### Analysis in Yeast

We used the yeast strain W303 P^R^ *mto1* (*MATα trp1-1 mto1::URA3*) [Colby et al., 1998]. *MTO1* was cloned in the centromeric vector pFL39 [Bonneaud et al., 1991] through PCR amplification of *MTO1* and digestion with *Sal*I and *Sac*I. The *mto1* mutant alleles were obtained by site-directed mutagenesis of a *MTO1* fragment [Ho et al., 1989], using suitable primers (Supp. Table S1). Mutant fragments were cloned in the *Ava*I and *Sac*I cloning sites of pFL39-*MTO1*. The *mto1* strain was transformed with pFL39 harboring wt or mutant *MTO1* alleles by lithium-acetate based methods [Gietz and Woods, 2002]. Respiratory activity and in vitro mt-DNA protein synthesis were performed as previously described [Barrientos et al., 2002; Goffrini et al., 2009]. Cytochrome *c* oxidase activity was measured according to Fontanesi et al., (2008) and Barrientos et al., (2009) on a mitochondrial-enriched fraction prepared according to Soto et al., (2009).

### Lentiviral Transduction

The wt *MTO1* cDNA was cloned into the pLenti6.3/V5-TOPO Vector (Invitrogen, Carlsbad, CA, USA), and virions were obtained as previously described [Zhang et al., 2009]. Mutant and wt fibroblasts were infected with viral supernatant and selected upon exposure to 2 µg/ml Blasticidin (Invitrogen).

## Results

### Molecular and Biochemical Analyses in Human Samples

By Sanger sequencing we screened *MTO1* in a cohort of mitochondrial defective patients with cardiomyopathy, and found that Pt1 was compound heterozygous for the previously described c.1402G>A/p.Ala428Thr mutation and for a novel missense substitution (c.1430G>A/p.Arg477His), whereas siblings Pt2 and Pt3 harbored a homozygous c.1232C>T/p.Thr411Ile change (Fig. 1A). By WES analysis on a second group of familial cases with CIV deficiency (see *Materials and Methods*) and no known genetic defect, we identified an additional case, Pt4, with the same homozygous c. 1232C>T/p.Thr411Ile change. Her clinically affected sister (Pt5) was shown to harbor the identical homozygous variant (Fig. 1A).

Spectrophotometric biochemical assays of the respiratory chain complexes activities revealed defects in CI, CIV, or both (Table 1). No clear evidence of CIII deficiency was obtained in any tissue sample of the present study, in contrast with a previous study [Ghezzi et al., 2012; #6 in Table 1]. A partial but significant reduction in MRR, SRC, and OCR/ECAR was measured by SeaHorse microscale oxygraphy in fibroblast cell lines of Pt1 and Pt2 (Supp. Table S2).

### In Silico Analysis

To test the potential deleterious effects of the p.Thr411Ile and p.Arg477His, we first used bioinformatic tools based on heuristic methods for predicting pathogenic variants. The prediction of a putative pathogenic change is based on evolutionary conservation, plus other features, such as predicted structural effects (Polyphen-2, MutPred), predicted functions (MutPred), and local sequence and gene ontology score (SNPs&GO). Both mutations scored a “probably pathological” prediction by each method (Supp. Table S3).

**Figure 2:**
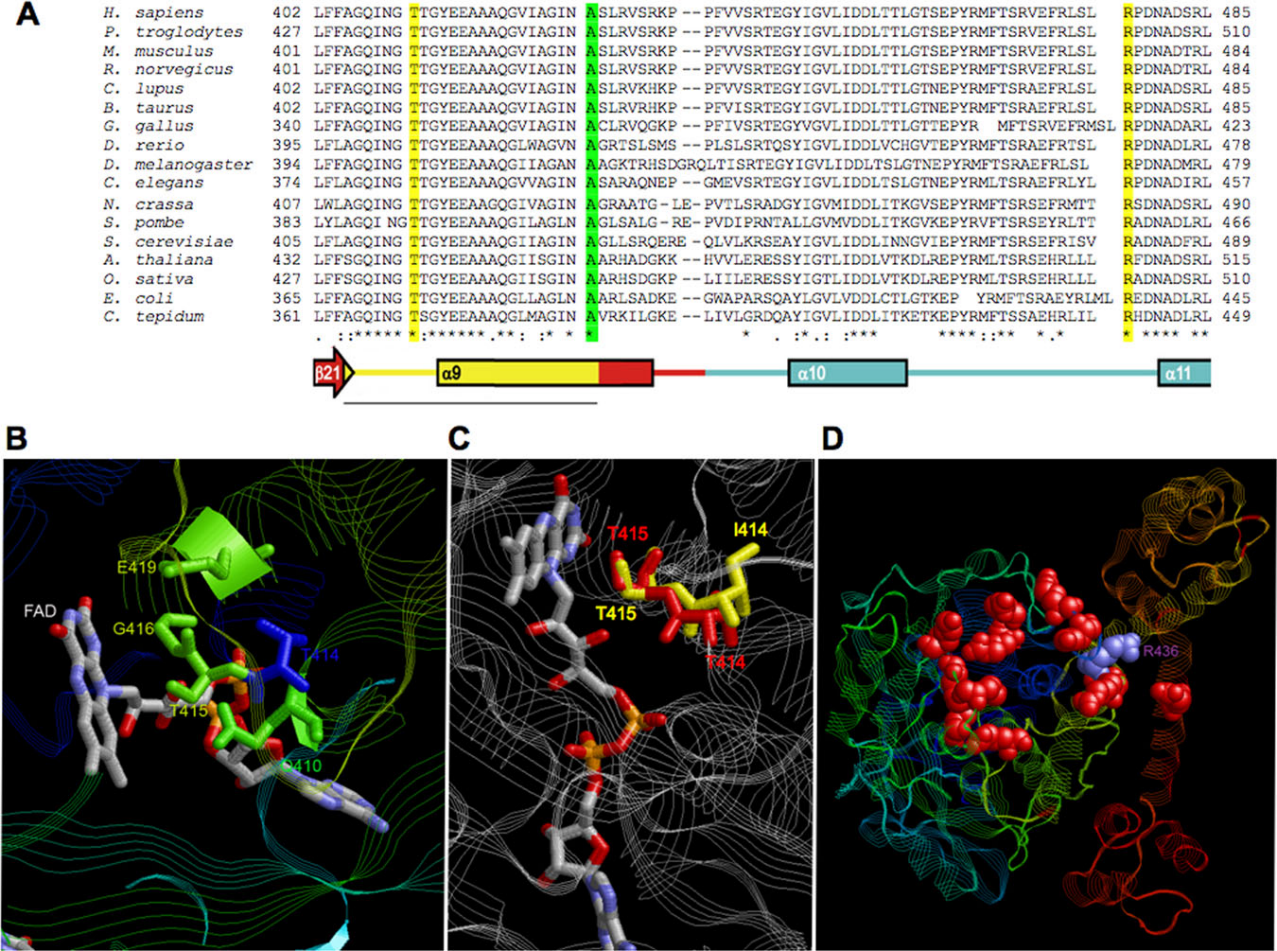
In silico structural analysis. A: Alignment of Mto1 proteins from animals, yeasts, plants, and eubacteria around mutated amino acids. In yellow the amino acids corresponding to mutations hThr411Ile and hArg477His, in green the amino acid corresponding to mutation Ala428Thr [Ghezzi et al., 2012]. The corresponding secondary structure elements for the *C. tepidum* GidA structure are indicated and colored according to Meyer et al. [2008]. B: Structure of the *C. tepidum* GidA region around the FAD moiety. Amino acids of the motif 2 of GidA which bind the FAD group are indicated. For simplicity, the numbers refer to the equivalent position in yeast Mto1. C: Structure of the *C. tepidum* GidA region around the FAD moiety superimposed to the model structure of GidA. The wt structure of the amino acids equivalent to threonines 414 (T414) and 415 (T415) in yeast Mto1 is in red. The predicted structure of the amino acids equivalent to yeast mutant isoleucine 414 (I414, corresponding to the human mutation Thr411Ile) and adiacent threonine 415 (T415) is in yellow. For simplicity, the numbers refer to the position in yeast Mto1. D: Overall structure of the *C. tepidum* GidA with basic amino acids (in red), which form a pocket who is predicted to bind the D-stem of the incoming tRNA. The bacterial Arg436 residue (R436), equivalent to hArg477 and yArg481, is in magenta.

We further tested the pathogenicity of these mutations on the basis of the structure of GidA, the eubacterial ortholog of Mto1. Its structure has been resolved in *Escherichia coli*, *C. tepidum*, and *Aquifex aeolicus* [Meyer et al., 2008; Osawa et al., 2009; Shi et al., 2009]. GidA contains four domains: an α/β FAD-binding domain; a small N-terminal insertion-domain 1; a large α/β insertion-domain 2, which binds NADH; a large α C-terminal domain, which contributes to the binding of the tRNA and promotes the dimerization with MnmE, the ortholog of Mss1/Gtpbp3. Human threonine 411 (hThr411) is part of motif 2, which is highly conserved from bacteria to eukaryotes (Fig. 2A) and is contained in the α/β FAD-binding domain. Motif 2 includes residues from the C-terminus of sheet β21, the loop between β21 and the residues from the N-terminal of helix α9. In *C. tepidum* GidA, this motif includes three conserved residues: Gln366 (hGln407, yeast Gln410), Gly372 (hGly413, yGly416), and Glu375 (hGlu 416, yGlu 419), plus Ser371, which is either conserved or substituted conservatively by threonine (e.g., hThr412, yThr415). The residues are all involved in FAD binding through interaction with the FAD ribitol and pyrophosphate moieties (Gln366 and Gly372) or with the isoalloxazine ring-containing active site (Ser371 and Glu375) (Fig. 2B). Moreover, the side chain of serine 371 is oriented to the central ring of isoalloxazine, suggesting a functional role in the catalytic process and/or binding/stabilization of FAD [Meyer et al., 2008]. As a matter of fact, substitution of Thr382 in *A. aeolicus* GidA, corresponding to Ser371 in *C. tepidum* GidA, results in inability of complementing the methylaminomethyl modification at position 5 of uridine (mnm^5^) during exponential growth of GidA-deficient *E. coli* [Osawa et al., 2009]. We hypothesized that the substitution of hydrophilic hThr411/yThr414 (corresponding to threonine at position 370 of GidA) with a hydrophobic, bulky isoleucine changes the position of the adjacent amino-acid residue (hThr412/yThr415 or bacterial Ser371). To support this hypothesis, we constructed a structural model in which the threonine at position 370 (yThr414) was changed to isoleucine. This change altered the orientation of the side chain of the adjacent amino acid and increased the distance relative to the isoalloxazine ring (Fig. 2C).

We identified the hArg477 residue in humans as equivalent to *C. tepidum* Arg436, which is located in a highly conserved loop between helices α10 and α11 in the C-terminal domain (Fig. 2A). Arg436 takes part in a cluster of several basic amino acids (Lys, Arg, and His), conserved in bacteria and eukaryotes, and predicted to form a positively charged pocket, which binds the phosphates of the D-stem backbone of the incoming tRNA (Fig. 2D) [Meyer et al., 2008; Osawa et al., 2009]. The substitution of the equivalent arginine with alanine in GidA of *E. coli* is known to decrease the efficiency of mnm^5^ modification [Shi et al., 2009]. Therefore, we hypothesized that substitution of the fully charged human Arg477 with the partially charged His477 could also decrease the affinity for the incoming tRNA.

### Analysis in Yeast

**Figure 3:**
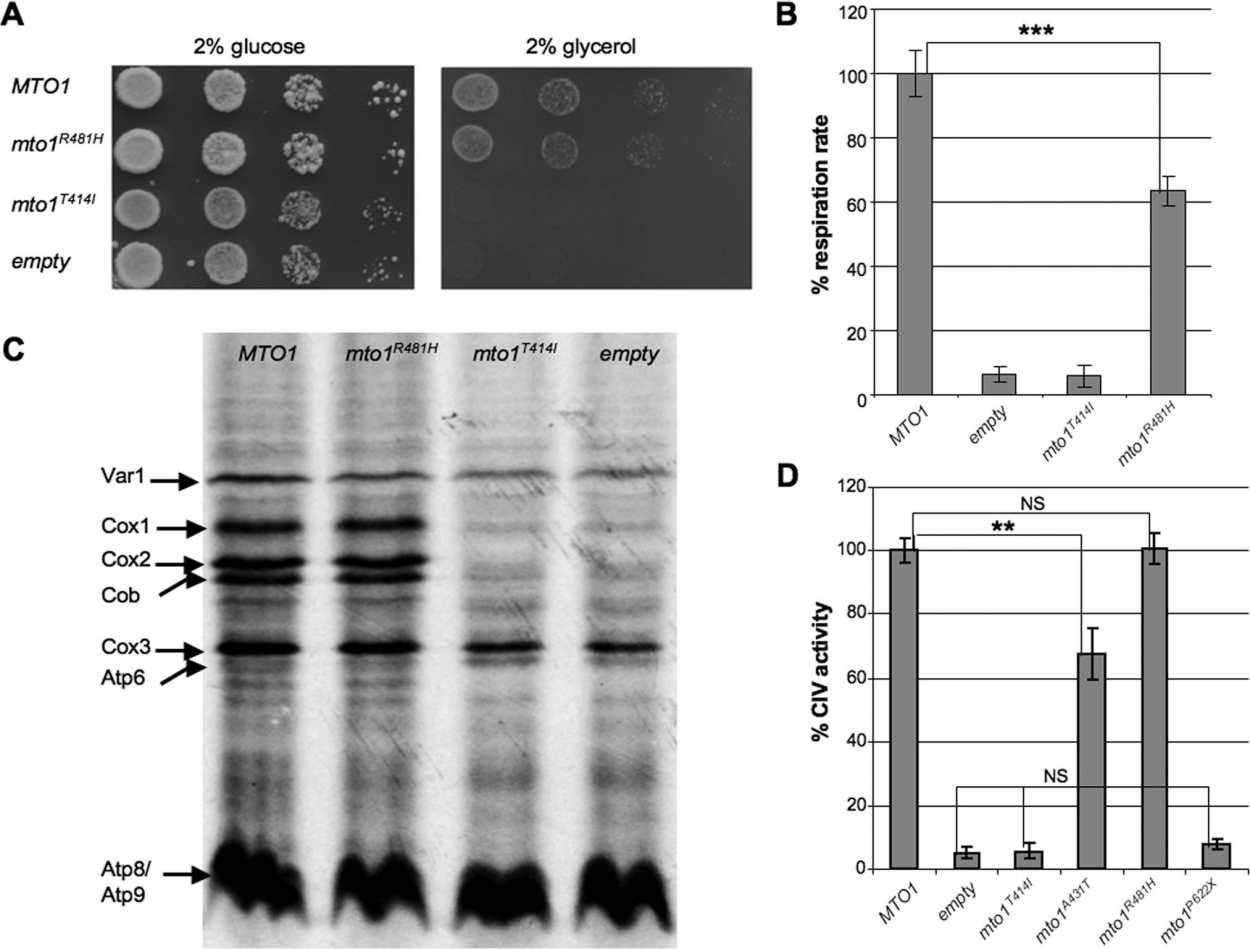
Yeast studies. A: Growth of *Δmto1* strain transformed with *MTO1* wt allele, *mto1^R481H^*, and *mto1^T414I^* mutant alleles or empty plasmid on YP medium supplemented with 2% glucose (left panel) or 2% glycerol (left panel). Cells were pregrown on YP+glucose and plated after serial dilutions to obtain spots of 5 × 10^4^, 5 × 10^3^, 5 × 10^2^, and 5 × 10^1^ cells/spot. Pictures were taken after 2 days of growth. B: Respiratory activity of *Δmto1* strains transformed with *MTO1* wt allele, *mto1^R481H^*, and *mto1^T414I^* mutant alleles or empty plasmid. Respiratory rates were normalized to the strain transformed with wt *MTO1*, for which the respiratory rate was 34.7 nmol min^−1^ mg^−1^. Values are the mean of three independent experiments, each with an independent clone. Two-tail, paired *t*-test was applied for statistical significance. ^***^P < 0.001. C: In vivo mitochondrial translation of *Δmto1* strain transformed with *MTO1* wt allele, *mto1^R481H^*, and *mto1^T414I^* mutant alleles or empty plasmid. Mitochondrial gene products were labeled with [^35^S]-methionine in whole cells in the presence of cycloheximide for 10 min at 28°C. Cox: cytochrome *c* oxidase; Cob: cytochrome *b*; Atp: ATP synthase; Var1: small mitochondrial ribosome subunit. D: Cytochrome *c* oxidase (CIV) activity of *Δmto1* strain transformed with *MTO1* wt allele, *mto1^T414I^*, *mto1^A431T^*, *mto1^R481H^*, and *mto1^P622X^* mutant alleles or empty plasmid. Cytochrome *c* oxidase activities were normalized to the strain transformed with wt *MTO1*, for which the activity was 368.8 units per mg of mitochondrial proteins. Values are the mean of three independent experiments, each with an independent clone. Two-tail, unpaired *t*-test was applied for statistical significance. ^**^P < 0.01.

To confirm the pathogenic role of p.Thr411Ile and p.Arg477His mutations predicted by in silico analysis, we introduced the corresponding mutant alleles (*mto1^T414I^* and *mto1^R481H^*) in the paromomycin-resistant yeast strain disrupted in *MTO1* (*Δmto1* P^R^ strain). The parental *Δmto1* P^R^ strain is unable to grow on oxidative carbon sources (Fig. 3A) [Colby et al., 1998]; the expression of *mto1^T414I^* mutant allele failed to correct this phenotype, whereas the expression of *mto1^R481H^* was able to restore oxidative growth, although to a lesser extent than wt *MTO1* (Fig. 3A). Accordingly, mitochondrial respiration was abolished in both *Δmto1* and *mto1^T414I^* strains, whereas it was restored by the *mto1^R481H^* strain to approximately 60% of the wt strain (Fig. 3B).

Since the p.Thr414Ile mutation is predicted to alter the position of the adjacent Ser371, which participates in the catalytic process involving FAD and/or in its binding/stabilization, we carried out a second set of experiments in the presence of increasing concentration of riboflavin (from 1 to 25 μM); however, neither defective oxidative growth nor reduced respiratory activity of the *mto1^T414I^* strain were rescued by riboflavin, suggesting that the catalytic activity of the mutant Mto1^T414I^ is fully impaired (data not show). An alternative explanation is that the mutant Mto1^T414I^ is unstable and quickly degraded; the absence of an antibody against the yeast Mto1 prevented us from evaluating the levels of Mto1 in different mutant strains.

To analyze the molecular consequences of the p.Thr414Ilr and p.Arg481His MTO1 mutations, we performed in vivo mitochondrial protein synthesis (Fig. 3C). As previously observed, we detected radiolabeled bands in the *Δmto1* strain corresponding to ribosomal protein Var1, cytochrome *c* oxidase subunit 3 (Cox3), and Atp subunits (Atp6, Atp8, Atp9), whose levels were similar to those of the *MTO1* strain. However, cytochrome *c* oxidase subunits 1 and 2 (Cox1 and Cox2) and cytochrome *b* (Cob) were absent. The *mto1^T414I^* strain behaved like the *Δmto1* strain, whereas *mto1^R481H^* was indistinguishable from wt *MTO1*, as previously observed for *mto1^A431T^*. Accordingly, CIV activity of *mto1^T414I^* and *mto1^P622X^* strains was indistinguishable from that of *Δmto1* strain (4%–7% relative to *MTO1* strain), it was 70% in *mto1^A431T^* relative to the wt, and identical to the wt in *mto1^R481H^* strains (Fig. 3D) (Supp. Table S4). In *mto1^T411H^* mutant strain, we measured the CI–CIII activity (following the reduction of cytochrome *c* in presence of NADH as electron donor and KCN as inhibitor of cytochrome *c* oxidase), to identify a possible explanation for the respiratory phenotype but no reduction was observed (data not shown).

### Complementation in Fibroblasts

To further confirm the pathogenic role of the milder mutation p.Arg477His, we analyzed the respiration in fibroblasts from Pt1, compound heterozygous for p.Arg477His and p.Ala428Thr, after transduction with a recombinant lentiviral construct expressing the wt *MTO1* cDNA. Infected cells were cultured in F14 medium, enriched in growth factors, to facilitate the recovery after infection and speed up cell growth. In our experience, these culturing conditions increase the values for SRC, an indicator of the bioenergetic reserve, in both control and mutant cells. Infected Pt1 fibroblasts showed marked increase of MRR (+146%) up to normal values. A mild MRR increase (+34%) was also observed in MTO1^wt^ cells (Supp. Fig. S1). These results support a causative role for both p.Arg477His and p.Ala428Thr MTO1 variants in defective mitochondrial respiration of Pt1.

## Discussion

A quite broad phenotypic spectrum was observed in *MTO1* mutant patients: from severe, rapidly progressive, ultimately fatal presentation in two compound heterozygous children for Arg620Lysfs*8 and Ala428Thr mutations [Ghezzi et al., 2012], to fulminant postnatal phenotype, or severe, but long-lasting, encephalo-cardiomyopathy in the two families with a homozygous p.Thr411Ile mutation (this work), to benign, compensated hypertrophic cardiomyopathy with modest neurological abnormalities in patients [Pt1 in this work; Pt3 in Ghezzi at al., 2012], bearing two missense mutations.

In silico analysis suggested potential pathogenic role for the missense *MTO1* mutations identified in our patients, but the yeast model allowed us to experimentally confirm their deleterious effects, dissecting the contribution of single allelic variants and giving an idea of the severity of each mutation. As summarized in Supp. Table S4, the severity of the yeast phenotype associated with *mto1* mutations is: yArg481His (hArg477His) < yAla431Thr (hAla428Thr) ≪  yThr414Ile (hThr411Ile) = yPro622* (hArg620Lysfs*8) ≈ *mto1Δ*. In particular, the behavior of *mto1^R481H^* mutant is intermediate between that of the *mto1^A431T^* mutant, and that of the *MTO1* wt allele as far as oxidative growth, respiratory activity [Ghezzi et al., 2012], and CIV activity are concerned. A moderate effect of the yArg481His substitution is in agreement with the observation that the Arg versus His change is electrostatically conservative, the equivalent Arg in GidA from *C. tepidum* being predicted to participate in a positively charged pocket, formed by several Arg, Lys, and His residues, that binds the phosphates of the D-stem backbone of the incoming tRNA. This was confirmed by the partial, but significant, reduction of oxygen consumption but virtually normal CIV and CI–CIII activities detected in the *mto1^R481H^* mutant strain. A defect of CV in the *mto1^R481H^* mutant is unlikely, owing to the presence of normal amount of Atp6, Atp8, Atp9, and the previous observation that yeast strains carrying mutations in ATP6, 8, or 9 display defective oxidative growth but normal respiratory activity [Dujon, 1981] or reduced respiratory activity due to an indirect decrease of CIV [Kucharczyk et al., 2009].

Both *mto1^T414I^* and *mto1^P622X^* alleles behave as the null allele as for oxidative growth, respiratory activity, mitochondrial protein synthesis [Ghezzi et al., 2012], and CIV activity, albeit it is unclear if this is due to instability or loss of function of the mutant protein.

In some patients with *MTO1* mutations, the clinical presentations seemed to depend on the genotype and partly to comply with the phenotypic observations in yeast. For instance, the presence of one allele expressing the p.Ala428Thr variant, which, in yeast, is of intermediate severity, is probably not sufficient to complement the defects caused by the variant Arg620Lysfs*8, which is functionally null. Contrariwise, patients homozygous for the p.Ala428Thr mutation [Ghezzi et al., 2012] or heterozygous with the less severe p.Arg477His mutation (Pt1 in this report), have milder symptoms, and are alive and relatively well at 20 and 14 years of age respectively, although both with compensated hypertrophic cardiomyopathy.

However, in spite of carrying the same mutant genotype (Thr411Ile), the disease course was very different for the patients of the two families presented in this article. Although Pt2 and Pt3 both had perinatal onset and died very early, Pt4 and Pt5 presented with the first symptoms after only a few months of life and yet have reached adolescence, being now 19 and 12 years old, respectively. This observation highlights the importance of genetics and environmental variations in modulating the phenotype in humans. It is tempting to speculate that, in addition to protection/risk genetic factors differentially expressed in the two families, the different outcome could be due to the different pharmacological intervention, which was merely supportive in the first family, whereas included timely correction of lactic acidosis in the second, following DCA administration. Although the number of reported *MTO1* mutant patients is very low, as a matter of fact all patients that survived beyond infancy and are still alive had chronic DCA treatment starting immediately after the clinical onset. DCA was remarkably effective on metabolic acidosis, suggesting that vigorous treatment of this life-threatening condition allows compensatory mechanisms to take place, which can mitigate the effects of hypertrophic cardiomyopathy. DCA administration should therefore be considered in *MTO1* mutant patients. In spite of these encouraging effects on survival, DCA treatment could not prevent the development of neurological symptoms associated with highly deleterious mutations such as the Thr411Ile in Pt4 and 5, suggesting that neurodegeneration can progress independently from the correction of the metabolic status if MTO1 function is severely impaired.

Given the role of MTO1 as an optimizer of mtDNA translation, *MTO1* mutations can be associated with any combination of MRC deficiency, from isolated CIV deficiency (Family 2 in this article) to combined CI + CIV deficiency, the most common biochemical signature observed in *MTO1* mutant cases, to combined CIV + CIII deficiency, as previously reported [Ghezzi et al., 2012].

A rather specific genotype/phenotype correlation has been reported for several mutant factors involved in mtDNA translation [Rotig, 2011], an observation that still requires a finer dissection of the pathomechanism. Hypertrophic cardiomyopathy seems to be the clinical hallmark of *MTO1* mutations, although in the present study most of the patients were preselected on the basis of cardiac symptoms. In addition to the heart, clinical/radiological signs of brain involvement were clearly present in several *MTO1* mutant patients. Interestingly, a recently reported patient, carrying p.Gly59Ala and p.Thr308Ala *MTO1* compound heterozygous changes, showed refractory infantile spasms and CIV deficiency, but no cardiac involvement [Vasta et al., 2012]. However, the pathogenic role of these very variants remains unproven and the c.922A>G/p.Thr308Ala is reported as a SNP (dbSNP: rs145043138) with a minor allele frequency of 0.3%.

Yeast strains harboring *mto1^A431T^* or *mto1^R481H^* mutant alleles did show no evident defects in mitochondrial proteins synthesis; this observation is concordant with the lack of obvious impairment in mtDNA translation found in Ala428Thr and Arg620Lysfs*8 mutant fibroblasts [Ghezzi et al., 2012], suggesting that the pathogenic effects of *MTO1* mutations are not due to reduced levels of mtDNA-encoded subunits of the respiratory chain. Likewise, mitochondrial protein synthesis was not reduced in cells carrying deleterious mutations of, or having been knocked down for, *MTO2/TRMU* [Sasarman et al., 2011]. It is possible that amino-acid substitutions, that is, qualitative alterations of the primary structure of mtDNA proteins, rather than quantitative decrease of global protein synthesis, may play a major pathogenic role in both MTO1 and MTO2 mutant cells. The 5-carboxymethylaminomethylation and the 2-thiolation of the wobble uridine increase the accuracy of translation when guanidine is the third base of Gln, Glu or Lys codons, and prevent codon-anticodon pairing when the third base is a pyrimidine [Kurata et al., 2008; Murphy et al., 2004; Yarian et al., 2002]. Accordingly, in Ala428Thr and Arg620Lysfs*8 compound heterozygous fibroblasts, mtDNA-dependent CI, CIII, and/or CIV showed reduced activity, in spite of quantitatively normal mitochondrial protein synthesis, suggesting that errors in translation can determine the synthesis of qualitatively altered CI, CIII, and CIV mtDNA-encoded subunits [Ghezzi et al., 2012]. Likewise, CIV activity was reduced in the *mto1^A431T^* yeast strain, although the total levels of Cox1, Cox2, and Cox3 were similar to those of *MTO1* wt. This hypothesis is testable, by systematic investigation of human or yeast mutant cells, through mass spectrometry and other proteomics approaches. Another possibility is that MTO1 may play a second role in mitochondria besides 5-carboxymethylaminomethylation of the wobble uridine, as previously reported for other enzymes, which modify tRNA in bacteria [Nicholson, 1999; Roovers et al., 2008] and, potentially, for MTO2 [Sasarman et al., 2011]. Alternatively, the 5-carboxymethylaminomethylation of the tRNA can have additional functions besides the optimization of mitochondrial translation, as hypothesized for the thiolation of the wobble position catalyzed by MTO2 [Sasarman et al., 2011].

This study confirmed that *MTO1* mutations are associated with a mitochondrial disorder, characterized by hypertrophic cardiomyopathy, lactic acidosis, and MRC deficiency, albeit with a broad range of severity and frequent involvement of brain, possibly depending on the treatment. Moreover, we showed that the use of a suitable recombinant yeast model can validate the pathogenic role of variants found in human patients.
