# Supplementary material for: MTO1 Mutations are Associated with Hypertrophic Cardiomyopathy and Lactic Acidosis and Cause Respiratory Chain Deficiency in Humans and Yeast
Source: Hum Mutat. 2013 Sep 17;34(11):1501–9. doi: 10.1002/humu.22393 (PMC4028987; doi:10.1002/humu.22393)
Supplement: Supplementary file 1 [file humu0034-1501-sd1.pdf]

SUPPORTING INFORMATION

Case reports

Patient 1

Patient 1 (Pt1) was born at 40 weeks of gestation as the first child of non-consanguineous Italian parents (Figure 1A). Birth weight was 3200g (50th percentile), length 52cm (50th percentile), head circumference 36cm (50th percentile), APGAR scores 9-10. On her second day of life the patient became apnoeic with severe metabolic acidosis and lactic acidemia (8-12mM n.v. <2). Metabolic work-up showed high plasma alanine (720µM; n.v. 180-400), and increased urinary lactate, pyruvate, and Krebs cycle intermediates. Metabolic acidosis failed to respond to intravenous sodium bicarbonate, thiamine and biotin, but markedly improvement was obtained by dichloroacetate (DCA 50 mg/kg/day initially, later lowered to 25 mg/kg/day), with dramatic reduction of plasma lactate (<3mM).

A muscle biopsy, taken at 15 days after birth, showed multiple defects of MRC complexes, with strong reduction (<5% residual activity) of CI and CIV activities. The child was discharged at the age of 1 month in good control of blood lactate levels (<3 mM) under chronic DCA treatment. The clinical course during the following years was complicated by feeding difficulties, failure to thrive and neurological symptoms, e.g. myoclonic seizures for the first years of life. Nowadays, aged 14 years, her weight is at the 10th percentile, neurological development is moderately delayed, with hypotonia, dystonia and poor speech. Several brain MRIs showed abnormal bilateral hyperintensities in the capsulae surrounding the claustra (Figure 1B). From the age of 8 years she has suffered of hypertrophic cardiomyopathy, particularly in the posterior wall of the left ventricle (6 mm, n.v. 4) with reduced systolic fraction (40%). At age 7 years, DCA treatment was stopped because of abnormalities of visual and brainstem evoked potentials and nerve conduction velocities. Subsequent metabolic follow-up revealed mildly elevated blood lactate but no further episodes of metabolic acidosis. A second muscle biopsy at 8 years again showed severe reduction of CI (14%) and CIV (27%) activities, whereas the other MRC activities were normal. Oxygen consumption, assessed through micro-oxygraphy in cultured fibroblasts, displayed significant reduction of MRR, SRC and OCR/ECAR whereas RCR, an index of mitochondrial OXPHOS coupling, was normal (Supp. Table S2).

Patient 2

Patient 2 (Pt2) was born at 37 weeks of gestation (birth weight of 2.38kg; 4th percentile) as the male first child of 1st cousin consanguineous Pakistani parents (Family 1; Figure 1A). On the first day of life, he developed severe poor feeding and mild hypoglycaemia and was admitted to the Special Care Baby Unit. Over the first 3 months of life, he developed hypotonia, his weight gain was poor and an echocardiography (performed at 3 months of age because of the detection of a cardiac murmur) demonstrated severe left ventricular hypertrophy with posterior wall thickness (8mm). Lactic acidemia was noted, with blood lactate varying between 9.5 and 14.6 mM. Metabolic work-up showed high plasma alanine (690μM, n.v. <400), and increased urinary lactate, 3-methylglutaconic acid and accumulation of Krebs cycle intermediates. A muscle biopsy, taken at 6 months of age, showed decreased staining for cytochrome *c* oxidase (COX) and severe deficiency in both CI and CIV activities (<10% residual activities). Brain MRI showed symmetrical, bilateral abnormal signals in fornices, globus pallidus, thalamus, subthalamic nucleus, substantia nigra, dorsal mesencephalon, pons and to a lesser extent dentate nuclei of the cerebellum (Figure 1C). A lactate peak was detected on [H^+]-MR Spectroscopy. The clinical course during the following months was complicated by persistent hypotonia and failure to thrive despite nasogastric feeding. At 12 months of age the child developed pneumonia associated with worsening metabolic acidosis and died of irreversible cardiorespiratory arrest. Oxygen consumption assessed by micro-oxygraphy in cultured fibroblasts displayed significant reduction of MRR, SRC and OCR/ECAR with normal RCR (Supp. Table S2). The mtDNA sequence was normal.

Patient 3

Patient 3 (Pt3), the younger brother of Patient 2, was born at 34 weeks gestation (birth weight of 2.17kg; 25th percentile). This child was born with hypospadias and an accessory digit at the base of the palmar aspect of the left thumb. In view of the family history, plasma lactate was monitored in the neonatal period and found to be elevated (7-10 mM). An echocardiogram at 1 month of age showed mild left ventricular hypertrophy with a posterior wall thickness of 7mm. His subsequent clinical course was complicated by feeding difficulties and failure to thrive. He was admitted for nasogastric tube feeding and no further invasive investigations were performed

at the family's wishes. He received palliative care and died suddenly at home at the age of 3 months.

Patient 4

Patient 4 (Pt4) was born at term as the first female child of 1st cousin consanguineous Pakistani parents (Family 2; Figure 1A). No feeding or respiratory difficulties occurred in the perinatal period. At three months of age, she developed severe metabolic acidosis with lactic acidemia associated with bronchiolitis-like illness. Metabolic work-up showed increased urinary lactate. A muscle biopsy, taken at 3 months of age, revealed decreased histochemical reactivity for COX and a severe CIV deficiency (<10% of controls), with CI activity reported as normal. Echocardiography demonstrated mild biventricular hypertrophic cardiomyopathy, which improved on serial scans over a number of years and did not require medication. The clinical course during the following years was complicated by speech and language delay, failure to thrive and recurrent hospital admissions with lactic acidosis associated with intercurrent infections with an admission to intensive care at age 2 years due to generalised seizures and encephalopathy. Nowadays, aged 19 years, her weight is <3rd percentile, her psychomotor development is mildly delayed and she is in special secondary education. Her menarche occurred normally, at 13 years of age. The frequency and severity of admissions has reduced gradually with age and her last acute admission was aged 8 years. She has persistent fatigue with chronic lactic acidosis (5.0-8.0mM) and Vitamin D deficiency for which she takes regular ergocalciferol and sodium bicarbonate supplements. DCA was used regularly from the age of 16 years with a reduction in resting plasma lactate levels to 4.0 mM but has now been stopped.

Patient 5

Patient 5 (Pt5), the younger sister of Patient 4 was born at term (birth weight of 2890g; 9th-25th percentile), with no perinatal respiratory or feeding difficulties. At five months of age the patient developed severe metabolic acidosis and lactic acidemia (>22.0mM), associated with an upper respiratory illness. Echocardiography demonstrated dilated cardiomyopathy with left ventricular hypertrophy and an electrocardiogram demonstrated a Wolff-Parkinson-White syndrome. Metabolic work-up showed high plasma alanine (695µM), and increased urinary lactate with ketonuria and dicarboxylic aciduria. A muscle biopsy, taken at 7 months of age, showed similar

findings to her sister, i.e. severe COX defect. She had a further severe decompensation aged 9 months associated with bronchiolitis and was ventilated for 4 weeks with repeated attempts at extubation failing due to rising lactic acidosis and worsening cardiomyopathy (fractional shortening 15%), with pericardial effusion. She required drug treatment for cardiac failure and her lactic acidosis was successfully treated with DCA at 50mg/kg/day with plasma lactate levels falling to 3.0 mM. The clinical course during the following years was complicated by psychomotor delay, increasing lower limb spasticity, failure to thrive (needing nasogastric feeding) and recurrent hospital admissions with lactic acidosis associated with intercurrent infections. Nowadays, aged 12 years of age, her weight and height are at <3rd percentile and psychomotor development is severely delayed. She is able to walk independently but has no speech and limited non-verbal communication; she is also in special education. The frequency and severity of admissions has reduced gradually with age and her last acute admission was at aged 11 years. Her cardiomyopathy has gradually improved with a fractional shortening of 30% and she takes only digoxin and lisinopril. Chronic DCA has been discontinued. Full mitochondrial DNA sequencing was negative in this family, as was the diagnostic screening of several COX assembly factor genes including *COX10* and *COX15*.

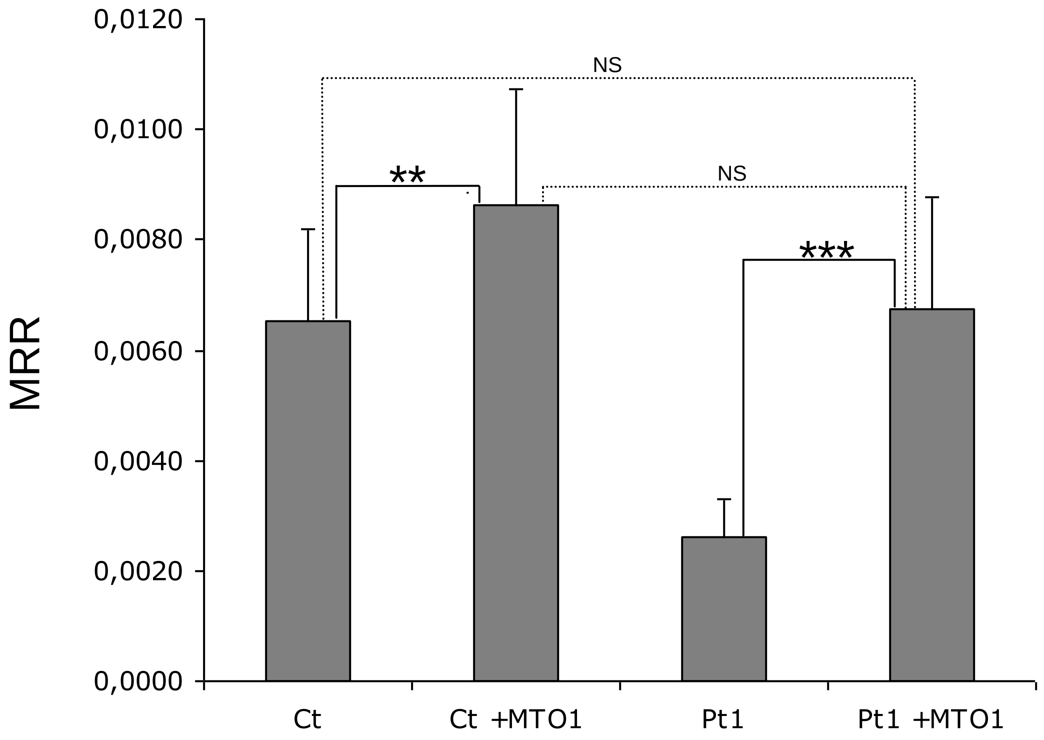

	Pt1			Ct		
	naive	+MTO1	Fold increase	naive	+MTO1	Fold increase
**MRR**	41	102	2.5	100	134	1.3
**SRC**	52	177	3.4	100	223	2.2
**RCR**	93	132	1.4	100	130	1.3

Supp. Figure S1. Complementation in fibroblasts. Maximal respiration rate (MRR), measured in fibroblasts from Patient 1 (Pt1) and a control subject (Ct), in naive condition or overexpressing MTO1 (+MTO1). MRR values are expressed as pMolesO_2/min/cells. Data are represented as mean ± SD. Two-tail, unpaired Student's t test was applied for statistical significance. ***: $p < 0.001$; **: $p < 0.01$; NS: not significant ($p > 0.01$).
The percentages of maximal respiration rate (MRR), spare respiratory capacity (SRC), respiratory control ratio (RCR) in patient 1 (Pt1) compared to a control subject (Ct), are listed in naive condition and after overexpression of MTO1^{wt} (+MTO1). The values of untreated control fibroblasts were considered as 100%.

Supp. Table S1. Oligonucleotides used in this work

**Human *MTO1* exon**	**Forward primer**	**Reverse primer**
1	TCCCTCACCAGGAAAGTAGCTC	CCCCGCTTCAGACCGG
2-3	GTAGTATATCTTTCACGTTTTCTATTTTTTATCAT	TCCTTGAATAAGCAACATATCTCCAC
4	ATTGCACCACTGCCCTCC	TTACAGTACTTCCTGCTGTG
5-6	TTAGTGTCTATTAAAGTCAGTACGTATCATGTG	TGACTGTAGCTAAGGCCTCCTCAC
7	TTTTTAAAGGGCATTTAAGGGTAATG	AGCCATCTCCAAACACCCTG
8	CAATACTTGCTTTCTTCCTGTCCC	AGTGTGGTTTTTAAAGCAAATTTTTTT
9	CAAGAGGATCACTTGAGGCCA	AACGGATTTTGAAAAGAAGCCAA
10	TCTTTTGGTATTTATTCTGAGATCTGATATTAT	TGTAGGTGTTGCAATGCTCTTAGC
11	AAAGGGAACACCTACCCAAC	GGCCATTATAACCCCAGGTT
12	TCAGTTTCCCATTTGTAAAATGAAGA	GTTCAGTTTTAGGTTCACTGTCTCTCTC
**Yeast *MTO1***	**Forward primer**	**Reverse primer**
Cloning[a]	gggggGTCGACgcttactgccactattagtcacg	gggggGAGCTCcgacagtgagttgcccttttgc
Mutagenesis	cttcacttaaatcttgagggcatcc	gcagatctgttttcagcctgtgg
T414I mutagenesis[b]	gccggacaaataaatggtaTtacaggctacgaggaagccg	cggcttcctcgtagcctgtaAtaccatttatttgtccggc
R481H mutagenesis[b]	cagaattcagaatcagcgtaCATgccgataacgcagactcag	ctgaagtctgcgttatcggcATGtacgctgattctgaattctg

[a] In upper case the endonuclease restriction sites
[b] In upper case the bases which are changed to introduce the desired mutations

Supp. Table S2. Oxygen consumption and extra-cellular acidification measurements in fibroblasts from patients 1 and 2 (Pt1 and Pt2)

	Pt1 [a]		Pt2 [b]	
	% of Ct	t-test vs Ct	% of Ct mean	t-test vs Ct
**MRR**	61	$1.4*10^{-6}$	46	$2.7*10^{-18}$
**SRC**	59	$9.9*10^{-4}$	46	$3.6*10^{-9}$
**RCR**	86	0.13	96	0.93
**OCR/ECAR**	40	$2.1*10^{-3}$	26	$6.9*10^{-9}$

Values are reported as percentages of the controls' (Ct) mean; unpaired two-tail Student's t-test was used for comparison between patients and controls. Measurements were performed in a Seahorse XF96 ([a]) or XF24 ([b]) instrument.

Supp. Table S3. In silico prediction of pathogenicity for *MTO1* mutations

	**SIFT**	**Polyphen-2**	**PANTHER**	**SNPs&GO**	**MutPred**
**hThr441Ile (yThr414Ile)**					
**Prediction**	Damaging	Probably damaging	0.95106/1	Disease	0.892/1
**Confidence/Reliability**	Low confidence		High	5/10	
**hArg477His (yArg481His)**					
**Prediction**	Damaging	Probably damaging	0.93769/1	Disease	0.710/1
**Confidence/Reliability**	Low confidence		High	4/10	

Supp. Table S4. Severity of the phenotypes associated with *Mto1* mutations in yeast

**Human mutation**	**Yeast mutation**	**Yeast phenotype**[b]				
		**Oxidative growth**	**Respiratory activity**	**Mitochondrial protein synthesis**	**CIV activity**	**Severity**
Arg477His	Arg481His	↓	↓	=	=	less severe
Ala428Thr	Ala431Thr	↓↓	↓↓	=	↓	intermediate
Thr411Ile	Thr414Ile[a]	↓↓↓	↓↓↓	↓↓↓	↓↓↓	most severe
Arg620Lysfs*8	Pro620* [a]	↓↓↓	↓↓↓	↓↓↓	↓↓↓	most severe

[a]: Thr414Ile and Pro620* behave as the null mutation *mto1Δ*.
[b]: “=” indicates that the phenotype is the same compared to *MTO1* wt, “↓↓↓” indicates that the phenotype is the same compared to the null *mto1* allele
